# Efficacy of pelvic-abdominal shields in radiation protection of patients undergoing radial coronary angiography: experimental analysis and recommendations for radiology practice

**DOI:** 10.1590/0100-3984.2025.0040-en

**Published:** 2025-12-08

**Authors:** Luciana Aparecida Salgado Rodrigues, Letícia Lucente de Campos Rodrigues, João Ricardo Antunes Marcos, Desidério Favarato, Maria de Fátima de Andrade Magon, Isabel Alves de Barros Morales, Erlon Oliveira de Abreu-Silva, George César Ximenes Meireles

**Affiliations:** 1 Hospital do Servidor Público Estadual de São Paulo (HSPE), São Paulo, SP, Brazil; 2 Instituto de Pesquisas Energéticas e Nucleares (IPEN/CNEN), São Paulo, SP, Brazil; 3 Instituto do Coração do Hospital das Clínicas da Faculdade de Medicina da Universidade de São Paulo (InCor/HC-FMUSP), São Paulo, SP, Brazil; 4 Sapra Landauer Serviço de Assessoria e Proteção Radiológica, São Carlos, SP, Brazil; 5 Hospital Beneficência Portuguesa de São Paulo, São Paulo, SP, Brazil

**Keywords:** Radiation protection, Radiation, ionizing, Radiometry, Radiation dosage, Radiology, interventional., Proteção radiológica, Radiação ionizante, Dosimetria, Doses de radiação, Radiologia intervencionista.

## Abstract

**Objective:**

To develop and evaluate the use of radiation shields for patients undergoing coronary angiography via the radial approach.

**Materials and Methods:**

Two pelvic-abdominal shields were developed-one for the posterior region and one for the anterior region. To analyze the entrance dose and its attenuation through the patient until reaching a detector as residual radiation, two dosimeter strips (right and left) were created and inserted into a phantom.

**Results:**

Comparing the shielded and unshielded groups, we found that the radiation doses at all detector positions were significantly higher in the shielded group (*p* < 0.0001).

**Conclusion:**

The use of pelvic-abdominal radiation shields made with 0.5 mm of lead is not recommended for patients undergoing interventional cardiology procedures, because it significantly increases radiation exposure and therefore does not comply with the As Low as Reasonably Achievable principle.

## Abstract (pt)

**Objetivo:**

Desenvolver e avaliar a utilização de anteparos de proteção radiológica para pacientes submetidos a coronariografia realizada por via radial.

**Materiais e Métodos:**

Foram desenvolvidos dois anteparos pelvicoabdominais, um para ser utilizado na região posterior e o outro na região anterior (manta). Para análise da dose de entrada e sua atenuação ao atravessar o paciente até a radiação residual alcançar o detector, foram desenvolvidas duas réguas dosimétricas (direita e esquerda) e um objeto simulador.

**Resultados:**

Ao se comparar o grupo sem anteparos e o grupo com anteparos, observou-se que em todas as amostras o grupo com anteparos apresentou valores maiores de dose em relação ao grupo sem anteparos (*p* < 0,0001).

**Conclusão:**

A utilização dos anteparos radiológicos pelvicoabdominais, confeccionados com 0,5 mm de chumbo, não se aplica a pacientes submetidos a procedimentos de cardiologia intervencionista, uma vez que não promove o princípio *As Low as Reasonably Achievable* ao aumentar, significativamente, a exposição à radiação.

## INTRODUCTION

Among the radiological modalities that use ionizing radiation, image-guided interventional cardiology procedures are those that most expose patients to high doses of primary radiation^**(^1^,^2^)**^. In interventional radiology, two types of exposure occur: occupational exposure, received by professionals; and clinical exposure, received by patients. These exposures differ significantly in the quantity and intensity of the energy transmitted. However, great attention should be paid to the clinical doses, because they are the source of the occupational exposures. In these two types of exposure, stochastic and deterministic (tissue reaction) effects can both occur^**(^2^-^30^)**^. Therefore, we hypothesized that the use of pelvic-abdominal shields over anatomical regions that do not interfere with imaging could reduce unnecessary patient exposure, promoting greater radiation safety during radial coronary angiography.

The guidelines established by the Brazilian Health Regulatory Agency, in Article 61 of Collegiate Board Resolution no. 611/2022, recommend the use of protectors with shielding equivalent to at least 0.5 mm of lead to protect radiosensitive organs, as long as they do not impair image quality or increase the dose required. The guidelines also emphasize dose control and management, following the As Low as Reasonably Achievable (ALARA) principle, to maximize benefits and minimize risks. The dose-limiting principle does not apply to patients; the focus is on optimizing doses for cost-effectiveness, with the aim of maximizing benefits, minimizing risks, and avoiding the occurrence of radiation-induced tissue reactions^**(^2^,^4^,^10^)**^.

The novelty of the present study is related to the creation of a systematized protocol for the use of a lead pelvic-abdominal shield that adheres to the ALARA principle. Therefore, the objective of the study was to develop and evaluate the use of pelvic-abdominal shields for the radiation protection of patients, to reduce the areas exposed to radiation during coronary angiography via the radial route, and to determine the impact that such protection has on the dose related to clinical, occupational, and procedure room exposures.

## MATERIALS AND METHODS

In this study, pelvic-abdominal prototypes were developed for the radiation protection of patients undergoing coronary angiography at the Vascular Interventional Radiology Clinic of the Institute for Medical Treatment of Francisco Morato de Oliveira Hospital for State Civil Servants, in the city of São Paulo, Brazil. Graduated polymethyl methacrylate (PMMA) dosimeter strips were used in a phantom to analyze the attenuation of ionizing radiation doses received by the patient. The study was approved by the local research ethics committee (Reference no. 3.983.030e), and all participants gave written informed consent to be involved in simulations that employed ionizing radiation, all of which were performed by the same physician (operator).

For the sample size calculation, we considered the number of groups irradiated during linearity testing of photoluminescent dosimetry systems, which suggested that five groups be irradiated and evaluated with *n* detectors. The number of samples was doubled, and 10 irradiations were performed in each group-with and without a shield-resulting in a total of 20 irradiations. Each irradiation used 22 optically stimulated luminescence detectors, of which 16 were nanoDot detectors (Sapra Landauer Serviço de Assessoria e Proteção Radiológica Ltda., São Carlos, Brazil) used for clinical dosimetry, three were InLight detectors (Sapra Landauer) used for occupational dosimetry, and three were InLight detectors (Sapra Landauer) used for procedure room dosimetry. The manufacturer provided technical support during the development of the research by supplying the detectors and performing the dose readings. These detectors are calibrated for X-ray readings according to the parameters required for clinical dosimetry (absorbed dose), given in milligrays (mGy), and occupational dosimetry (effective dose), given in millisieverts (mSv), also considering the backscatter of ionizing radiation.

Because this research was conducted on a phantom, the inclusion and exclusion criteria for patient data did not apply. However, inclusion was based on fixed data during the simulations: field size of 39 cm; table height of -14 cm; and seven projections per irradiation, with angles according to the institutional protocol.

Data were analyzed to determine whether the assumptions of normality and homoscedasticity were satisfied. The Shapiro-Wilk test was applied to assess normality, and Levene’s test was applied to assess the homogeneity of variances (Table 1). However, for most detector positions, the assumption of normality was not satisfied at the 5% significance level. Descriptive statistics for the radiation dose variable were calculated according to shielding condition and detector position. The data are presented as mean and standard deviation or as median. Comparisons between groups (with and without shielding) were performed using the nonparametric Mann-Whitney-Wilcoxon test.

**Table 1:** Normality and homogeneity test results.

Detector position	Shapiro-Wilk test		Levene’s test
	Shielded group	Unshielded group	
Right back	0.011	0.000	0.046
Left back	0.475	0.576	0.101
Anteroinferior	0.139	0.011	0.402
Posteroinferior	0.246	0.006	0.184
1st ledge on the right	0.902	0.574	0.007
2nd ledge on the right	0.097	0.723	0.000
3rd ledge on the right	0.577	0.709	0.007
4th ledge on the right	0.010	0.080	0.776
1st ledge on the left	0.045	0.619	0.073
2nd ledge on the left	0.681	0.037	0.004
3rd ledge on the left	0.663	0.434	0.043
4th ledge on the left	0.289	0.292	0.000
Below the table	0.219	0.036	0.080
Above the blanket	0.058	0.011	0.205
Flat panel, center	0.043	0.060	0.173
Flat panel, side	0.052	0.473	0.093

**Figure 1:**
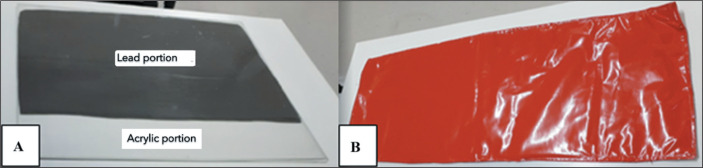
A: Lead shield for the posterior abdomen. B: Lead blanket for the anterior abdomen.

The posterior abdominal lead shield (Figure 1A) was made of 3-mm-thick PMMA, double-folded in a “wallet” shape, measuring 65.0 × 65.0 cm. Within this base, two 0.25-mm lead sheets, each measuring 65.0 × 42.5 cm, were inserted. The posterior shield was designed to be inserted between the procedure table and the mat upon which the patient lies. The blanket (Figure 1B) was made of washable canvas with two 0.25-mm lead sheets, each measuring 45.0 × 110.0 cm. The two shields together function as a barrier equivalent to 0.5 mm of lead.

**Figure 2:**
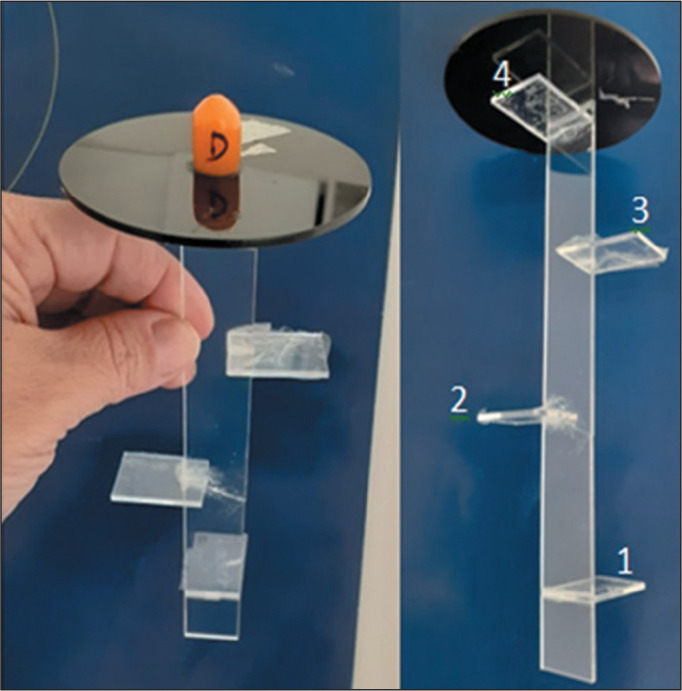
Image of one of the PMMA dosimeter strips.

Each PMMA dosimeter strip (Figure 2) had an overall length of 20 cm, with four 1.5 × 2 cm horizontal ledges, one every 5 cm, on which the four corresponding detectors were positioned. The strips were inserted into the nipple regions of the phantom. To better understand the dose reduction as a function of depth and the potential areas impacted by radiation, the radiation reduction along the trajectory of each strip was illustrated. The means of the variables collected at each level were represented in two axial computed tomography images of the chest, one corresponding to the group in which a shield was used (shielded group) and the other to the group in which no shield was used (unshielded group).

**Figure 3:**
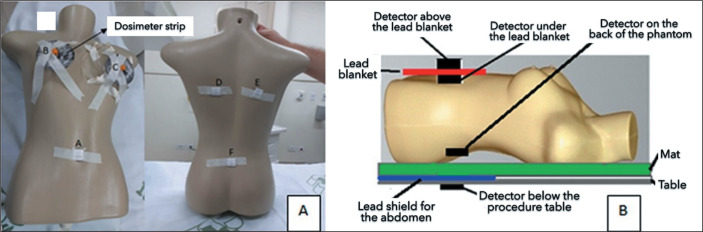
A: Distribution of nanoDot detectors in the simulation with shielding. Anteroinferior region (A), right PMMA dosimeter strip (B), left PMMA dosimeter strip (C), left posterolateral region (D), right posterolateral region (E), and posteroinferior region (F). B: Arrangement of the detectors in relation to the table, lead blanket (red), and posterior abdominal shield (blue)..

The phantom (Figure 3A) was developed from a polyurethane mannequin, with circumferences of 78 cm at the bust, 61 cm at the waist, 80 cm at the hip, and 15 cm around the shoulder, as well as a 40 cm width at the back, a shoulder-to-waist height of 37 cm, a waist-to-hip height of 15 cm, and a shoulder-to-hip height of 53 cm. The nipple regions were removed for the introduction of the dosimeter strips, and the phantom was filled with water. Figure 3A shows the distribution of the detectors, which allowed the verification of the incident radiation entering the skin until its attenuation as it passed through the phantom, resulting in residual radiation. Two detectors were positioned directly on the phantom, and eight were distributed along the two dosimeter strips. To verify the effectiveness of the pelvic-abdominal shield and the lead blanket, six detectors were positioned, as shown in Figures 3A and 3B: one under the procedure table, 5 cm from the upper end of the shield; one on the lead blanket; one under the blanket; three on the back of the phantom (in the right posterolateral region, left posterolateral region, and central dorsal region, respectively).

Two detectors with optically stimulated luminescence were fixed to a flat panel, one positioned in the center of the flat panel and the other 5 cm to the anterolateral region, to check the residual radiation on the flat panel.

The simulations, one to determine the positioning of the shields on the phantom and the others respecting the standard coronary angiography protocol, were performed in accordance with the following criteria: for the left coronary artery-right anterior oblique (RAO) 20° panoramic projection, 20° caudal/20° RAO, 35-45° cranial ± RAO, 35-45° cranial/20-40° left anterior oblique (LAO), and 25-45° caudal/20-40° LAO; and for the right coronary artery-30° LAO/± 30° caudal, 20-30° RAO. All simulations were performed in cine mode at 15 frames/s.

To monitor the occupational dose, three detectors were positioned on the physician: one on the left leg, one on the chest, and one on the left side of the skull (as a proxy for the lens of the eye). To monitor the environmental dose, a detector was positioned on each wall of the procedure room, 130 cm above the floor.

## RESULTS

This research was conducted from November 2019 to November 2020. During this period, 20 simulations were performed, 10 with lead screens and 10 without. After a reproducibility analysis, it was noted that two simulations in each group did not have the same values stipulated for the fixed technical parameters. Therefore, those two simulations were excluded, leaving eight simulations in each group, totaling 16 valid simulations, for analysis.

During the simulations, the technical parameters presented by the equipment showed that the voltage remained fixed at 77 kV with the use of an additional 0.1-mm copper filter in both groups, contributing to beam hardening by eliminating low-energy radiation. In the shielded group, the electron current ranged from 80 mA to 334 mA, whereas it ranged from 84 mA to 292 mA in the unshielded group. The cine time was 8.0 s in the unshielded group and 6.0 s in the shielded group. The highest doses were observed in the caudal LAO projections.

The *p*-values for the Shapiro-Wilk test and the Levene test, described in Table 1, demonstrate that in most positions the assumption of normality was not satisfied.

Table 2 shows that most positions were statistically significant, with *p*-values below 0.05. The median radiation dose was higher in the shielded group than in the unshielded group. Only three detectors (the one under the table, the posteroinferior one, and the one above the blanket) did not show statistical significance. Analysis of the detector data on the dosimeter strips showed that, regardless of the irradiated side, the doses received at the deeper ledges and at those closest to the back of the phantom were lower than were those received in the anterior region.

**Table 2:** Descriptive measures of the absorbed radiation dose, by detector position.

Position	Shielded group			Unshielded group			P-value†
	Median (mGy)	Mean (mGy)	Standard deviation (mGy)	Median (mGy)	Mean (mGy)	Standard deviation (mGy)	
Right back	1.61	3.10	2.59	0.49	0.63	0.48	0.004
1st ledge on the right^*^	1.88	1.96	0.87	0.48	0.52	0.23	0.001
2nd ledge on the right^*^	0.64	0.66	0.25	0.23	0.24	0.07	0.001
3rd ledge on the right^*^	0.40	0.42	0.13	0.17	0.17	0.05	0.001
4th ledge on the right^*^	0.17	0.18	0.04	0.09	0.10	0.02	0.001
Left back	5.12	4.82	1.94	1.89	1.81	0.64	0.003
1st ledge on the left^*^	3.68	4.16	0.99	1.35	1.32	0.12	0.001
2nd ledge on the left^*^	2.22	2.14	0.71	0.68	0.71	0.08	0.001
3rd ledge on the left^*^	0.68	0.71	0.22	0.32	0.32	0.06	0.001
4th ledge on the left^*^	0.58	0.60	0.25	0.26	0.27	0.06	0.001
Below the table	1.86	2.60	2.20	1.53	1.39	0.48	0.270^‡^
Posteroinferior	1.02	1.10	0.55	1.06	0.97	0.24	0.916^*^
Anteroinferior	0.08	0.09	0.02	0.06	0.01	0.01	0.008
Above the blanket	0.04	0.05	0.03	0.06	0.06	0.01	0.086^*^
Flat panel, center	0.19	0.20	0.08	0.12	0.13	0.03	0.008
Flat panel, side	0.18	0.28	0.20	0.11	0.12	0.04	0.018

* Dosimeter strip.

† Mann-Whitney-Wilcoxon test.

‡ Not statistically significant.

**Figure 4:**
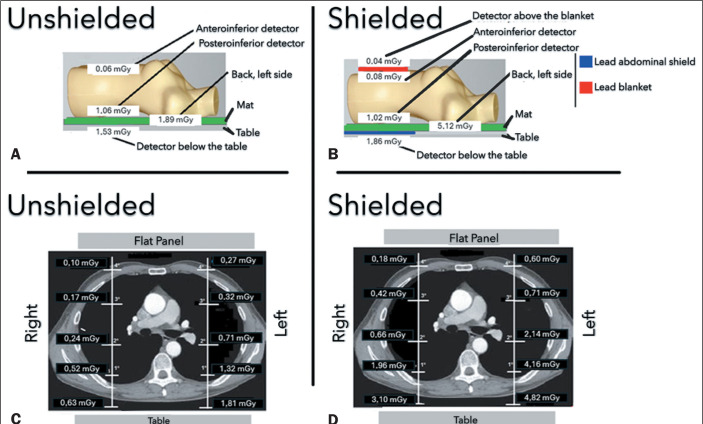
A,B: Median dose values showing the effectiveness of protection and the increase in dose with the use of shields in the incident air kerma dose, corresponding to the left lateral dorsum. C,D: Comparison of the mean dose values and their relationship with the extent to which they were attenuated.

For a better understanding of the results, Figure 4 shows the median dose values recorded directly in the phantom and the mean dose values obtained by the detectors positioned on the dosimeter strips, as visualized in the computed tomography images. The graphics compare the shielded and unshielded groups, demonstrating the spatial distribution of the dose in a linear profile for each condition analyzed.

Table 3 shows the doses recorded by detectors positioned on the physician and at locations around the procedure room. For most of the doses recorded, the values were higher in the shielded group than in the unshielded group.

**Table 3:** Accumulated doses received by the physician and the environment.

Position	Shielded group (mSv)	Unshielded group (mSv)
Physician		
Left leg	0.41	0.21
Chest	0.41	0.25
Lens of the eye	0.37	0.27
Environment		
Wall behind the C-arm	0.39	0.24
Right wall	0.36	0.21
Left wall	0.42	0.22
Table leg wall	0.35	0.35

## DISCUSSION

In this study, we have developed and evaluated the effectiveness of lead shields, as well as analyzing the correlations among the clinical doses, occupational doses, and doses delivered to the environment. Although we initially believed that the use of lead shields would result in dose protection and optimization by reducing the exposed areas during radial coronary angiography, their presence did not provide the expected protection, instead significantly increasing exposures in the regions studied, including the phantom, the physician, and the environment.

By analyzing the doses to the back of the phantom, dosimeter strips, and flat panel, we gained a better understanding of the attenuation behavior of the primary, secondary, backscattered, and residual radiation to which patients are exposed. In addition, we demonstrated the negative implications that the use of radiation shields has for clinical and occupational exposures, as well as its relationship with automatic exposure control systems and the environment in which the team operates during procedures.

Our data regarding the interaction between the energy of the primary beam and its attenuation show that the energy transferred was attenuated along its trajectory to the flat panel. In the shielded and unshielded groups, the left side received more radiation than did the right side, although the doses were higher in the shielded group. In the unshielded group, exposures were similar (84.0% on the right side and 84.5% on the left). In the shielded group, the right side attenuated 94.1% of the initial dose, compared with 87.5% for the left side. The mean doses were significantly higher in the shielded group: 392.06% higher on the right side and 166.35% higher on the left side. The mean values for exposures without a shield were compared with the values estimated in the consensus statement authored by Hirshfeld et al.^**(^28^)**^. In the present study, we considered the mean values obtained between the right and left sides without a shield, at the various sites-the back, the first ledge, the second ledge, the third ledge, the fourth ledge, and the flat panel-obtaining values of 100%, 75%, 39%, 20%, 15%, and 10%, respectively. In the consensus statement^**(^28^)**^, the estimated attenuation values (the absorbed dose was estimated by the inverse square law), for the left dorsal region, left posterior pulmonary region, posterior border of the heart, central cardiac region, middle mediastinum, anterior right lung, anterior thoracic wall, anterior subcutaneous tissue, and sternal skin, were 100%, 50.0%, 25.0%, 12.5%, 6.0%, 3.0%, and 1.5%; the flat panel was not considered. It is noteworthy that the dose detected at the flat panel is influenced by the primary beam and backscattered radiation, which, when colliding with the detector, can be reflected back toward the patient, thus increasing the absorbed dose.

The actual values were measured at depth along the trajectory, providing a better understanding of the effects that the dose can have on tissues. In addition, those values demonstrate that the use of high-density devices directly on the patient increases the doses. Our data are highly relevant, given that the dose of radiation received during complex procedures can be equivalent to radiotherapy doses, potentiating tissue reactions. The higher the doses are, the more pronounced are the lesions in the deeper layers. According to Leyton et al.^**(^2^)**^, it can take 13-21 months for lesions to manifest after exposure to radiation, and that interval may vary depending on the dose, the type of tissue irradiated, the procedure performed, and the radiosensitivity of the patient.

The comparison of doses in the anteroinferior region showed the median was 26.1% higher in the shielded group, whereas the values in the unshielded group were, on average, 33% lower. These results suggest that the lead blanket was not only ineffective in providing protection but also contributed to greater dose retention in the phantom. Previous studies have generated mixed results. Marcusohn et al.^**(^22^)**^ detected a slight increase in patient exposure with the use of a shield, whereas Kadish et al.^**(^27^)**^ reported that shield use minimized the radiation dose, without an increase in scattered radiation. Those differences can be attributed to differences between the two studies in terms of the materials used and the methods applied. Gutierrez-Barrios et al.^**(^29^)**^ noted that placing radioprotective drapes within the imaging field “may trigger an automatic increase in dose rate, significantly increasing patient dose”, as was observed in our study.

The comparison of the doses received at the detectors in the region of the posteroinferior abdominal lead shield did not reveal a statistically significant difference between the shielded and unshielded groups. However, when observing the median values for the detectors under the table, we found an attenuation of 30.72% in the unshielded group, compared with 45.16% in the shielded group. The doses received at the detectors in that position were 3.77% higher in the unshielded group than in the shielded group. However, the median entrance dose to the left back of the phantom (within the primary beam) was significantly (170.9%) higher in the shielded group.

When the effectiveness of this combined protection (i.e., the influence that the pelvic-abdominal shield and the lead blanket had on the radiation dose) is analyzed, it is worth noting that the use of the shields had a paradoxical effect, given that it increased the dose in the phantom. Although the shields attenuated a small portion of the dose, the expected level of radioprotection did not occur. Instead, the automatic exposure control system increased the intensity of the X-ray beam, increasing the entrance dose within the primary beam. Meanwhile, the blanket retained some of the radiation, which, upon impacting the blanket, reflected back onto the phantom, increasing the dose received by the phantom. In other words, neither type of protection conformed to the ALARA principle, demonstrating that the applicability of such protection should be studied for each radiological modality, given that what protects in certain circumstances can result in an increased dose in others.

In the present study, the use of lead shields on a phantom promoted an increase in the radiation doses, of 37.03% in the lens of the eye region, 64.0% in the chest region, and 95.0% at the left leg of the operator. When comparing the dose equivalent of the operator and the environment, we found that the exposures were 69% higher in the shielded group. That finding is contrary to what was reported in the studies of Osherov et al.^**(^17^)**^, Marcusohn et al.^**(^22^)**^, Lange et al.^**(^23^)**^ and Ordiales et al.^**(^24^)**^, the differences probably being due to the methods employed in those studies, in which shields made of other types of materials were used.

In our unshielded group, the area behind the C-arm was the most exposed to secondary and backscattered radiation in the air. In our shielded group, the highest doses were recorded on the left wall, where interventional physicians, echocardiographers, anesthesiologists, and the rest of the multidisciplinary team are typically positioned during most procedures. In both groups, the lowest doses were recorded on the right wall. The overall mean of all doses was highest in the shielded group. The dose was 38.5% higher on the wall behind the C-arm, 48.0% higher on the left wall, and 42.0% higher on the right wall.

Lead shields are designed to provide radioprotection. This study demonstrated that the use of an abdominal shield resulted in a 170.9% increase in the primary beam dose, whereas the blanket retained 26.1% of the dose in the phantom. This is especially concerning in interventional procedures involving pregnant patients, because mother and baby may both be exposed to more radiation. The use of lead shields should be carefully evaluated, given that high-density materials can scatter radiation, causing equipment with automatic exposure control to increase the dose, resulting in higher doses being received by the physician and the patient.

Our study has some limitations. First, we did not perform any tests using only the lead blanket on the phantom, without the presence of the pelvic-abdominal shield under the mattress. An isolated evaluation of the blanket would have allowed us to measure its true retention capacity for scattered radiation, which is especially important in clinical settings where interventional procedures are required for pregnant patients, in whom protection of the abdominal and pelvic regions is crucial to reducing fetal risk. Nevertheless, the presence of the blanket results in significant radiation retention in the phantom, as demonstrated in our results.

## CONCLUSION

This work highlights the importance of reviewing radiation protection protocols in interventional radiology and validating protective equipment before its clinical application. The indiscriminate use of protective barriers can, paradoxically, negatively impact patient dose absorption, contradicting the fundamental principles of radiation protection, such as the ALARA principle. Therefore, we conclude that the use of pelvic-abdominal radiation shields made of 0.5 mm lead is not appropriate for patients undergoing interventional cardiology procedures, because it increases radiation exposure significantly, thus failing to conform to the ALARA principle.

## Data Availability

Datasets related to this article will be available upon request to the corresponding author.
